# Relationship between fundus of the eye examination and arterial stiffness

**DOI:** 10.3389/fcvm.2024.1516787

**Published:** 2025-01-06

**Authors:** Eduardo Costa Duarte Barbosa, Ana Carolina Storch Klein, Julia Gabriela Storch Klein, Camila Samrsla Möller, Eliane Fátima Manfio, Bruna Eibel, Carolina da Silva Mengue

**Affiliations:** ^1^ Department of Hypertension and Cardiometabolism, São Francisco Hospital, Santa Casa de Misericórdia de Porto Alegre, Porto Alegre, Brazil; ^2^ School Medicine, Feevale University, Novo Hamburgo, Brazil; ^3^ Institute of Cardiology, University Foundation of Cardiology, Porto Alegre, Brazil; ^4^ Department of Ophthalmology, Ivo Correa-Meyer Institute, Porto Alegre, Brazil

**Keywords:** arterial stiffness, pulse wave velocity, fundus of the eye, cardiovascular diseases, hypertension

## Abstract

This review addresses the correlation between arterial stiffness, measured by pulse wave velocity (PWV), and retinal microvascular changes, highlighting the retina as an important accessible window for inferences about cardiovascular health. Arterial stiffness, intrinsically linked to vascular aging and several comorbidities, results in damage to the microcirculation, including ocular vasculature, which can act as a predictor of cardiovascular and cerebrovascular outcomes. The review highlights the relationship between PWV assessment and funduscopic examination, with the aim of improving diagnostic accuracy and optimizing the clinical application of these tools in the management of cardiovascular and ophthalmological diseases, thus promoting more effective and early intervention.

## Introduction

Changes in arterial stiffness are strongly associated with age, its main determinant, as well as with cardiovascular conditions and comorbidities, such as obesity, systemic arterial hypertension and diabetes mellitus (1, 2). Pulse wave velocity (PWV), in addition to other vascular parameters, becomes one of the forms of its assessment, being a well-established biomarker in the stratification of cardiovascular risk and in the identification of subclinical lesions (2). Carotid-femoral pulse wave velocity (cfPWV) is currently considered the gold standard for measuring the stiffness of large arteries. There is a large body of evidence that the assessment of the stiffness of large arteries can be clinically useful in hypertensive patients and, due to its relationship with age, is considered a key element in the assessment of vascular aging (3).

The coexistence of ocular changes, especially in the retinal microcirculation, may become frequent in these patients, representing another potential marker of vascular damage and severity of global cardiovascular risk (1, 2, 4). Given the close connection between the cardiovascular system and retinal microcirculation, the retina is often considered, by several studies, as an “accessible window for inferences”, since changes in retinal microcirculation are directly related to changes in the cardiovascular system (5, 6).

The aim of this review is to correlate the impact of arterial stiffness on fundus examination assessment and its clinical implication.

### Arterial stiffness

**Figure 1:**
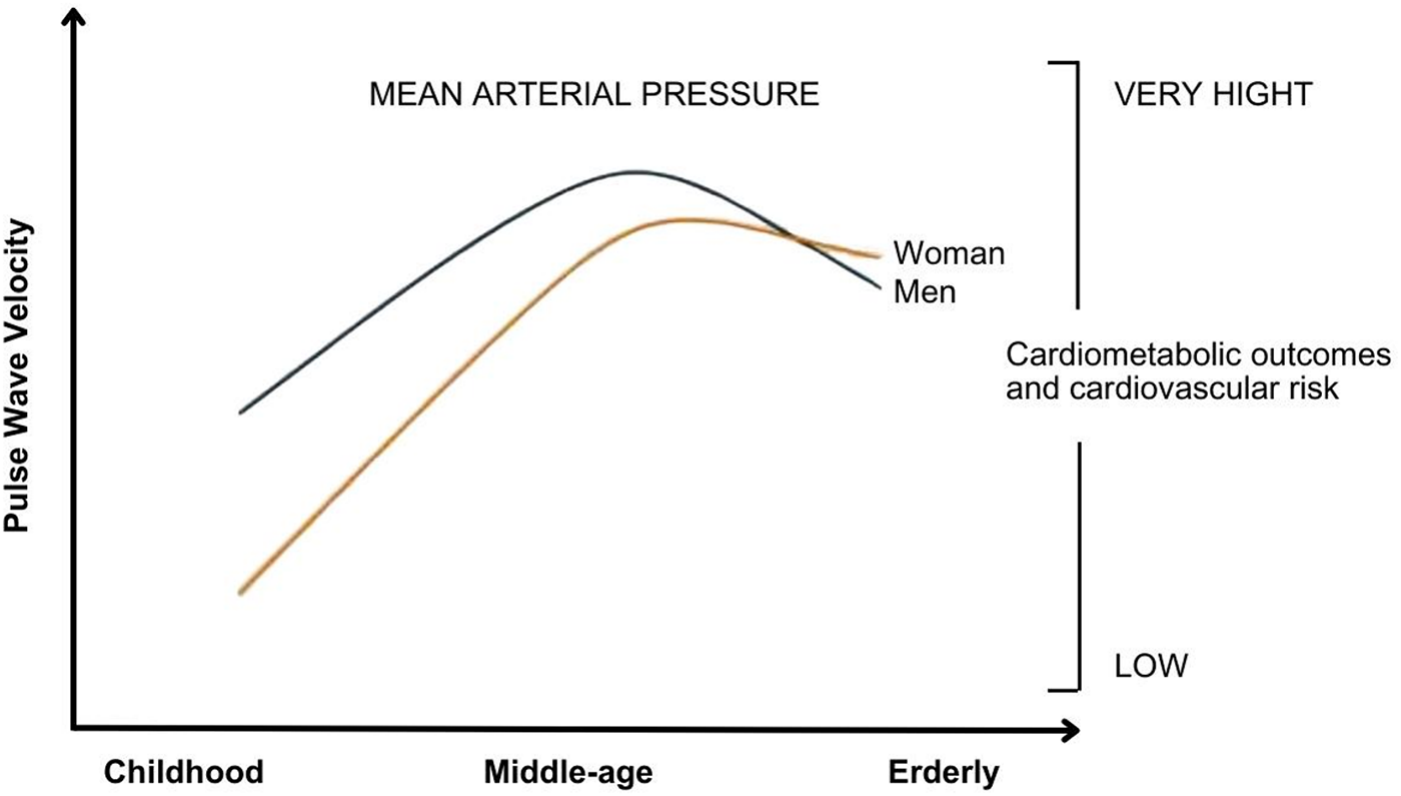
Relationship between mean arterial pressure behavior, in both sexes, with pulse wave velocity and cardiometabolic outcomes. It is observed that the increase in mean arterial pressure correlates with an elevation pulse wave velocity and a worsening in quality of life. Adaptive from: Ji H et al. (9).

Stiffness of large arteries, which develops with human aging and various pathological conditions, results in excessive transmission of pulsatile energy to the microcirculation of target organs, leading to subsequent damage to these structures (7, 8). In parallel, the reduction in lumen diameter and rarefaction of small arteries, common in accelerated vascular aging and in conditions such as essential hypertension, metabolic syndrome, and diabetes, increase total peripheral resistance and mean arterial pressure, which in turn contribute to greater stiffness of large arteries (7, 8). It is suggested that there is an “interaction” between large and small arteries, indicating a strong interdependence between changes in these two vascular structures (7). Furthermore, it is known that BP values progress more rapidly in women than in men, and women catch up with men in middle age in the extent of potentially significant vascular disease (Figure 1) (9). Furthermore, arterial stiffness tends to increase with human aging and is associated with an elevated risk of adverse outcomes in both short- and long-term follow-ups. Thus, arterial stiffening is associated with an increased risk of cardiometabolic outcomes such as hypertension and diabetes, as well as chronic kidney disease, cardiovascular disease and its components, including coronary heart disease, heart failure, stroke, and death (10).

### Pulse wave velocity (PWV)

Pulse Wave Velocity (PWV) is defined as the speed at which the pressure wave generated by ventricular ejection travels between two points in the arterial system, measured in meters per second (11). Recognized as the gold standard for assessing arterial stiffness, PWV stands out for being a simple, accurate, reproducible method with high predictive value (11–13). In clinical research, the reference method is carotid-femoral PWV (cfPWV). The cfPWV is calculated by dividing the distance traveled by the time. Validated methods available include those that use pulse tonometry and piezoelectric and oscillometric mechanotransducers (14). PWV measurements vary according to factors such as age, sex, blood pressure, ethnicity, and even the measuring devices and techniques used (15).

Although indirect arterial tonometry is widely used to obtain cfPWV, the oscillometric method, which measures blood pressure in the brachial artery, offers a simplified, reproducible, and equally reliable alternative (11). In addition to its correlation with the presence and extent of atherosclerosis in coronary, cerebral, and carotid arteries, PWV has a predictive value for cardiovascular disease that goes beyond traditional risk factors, both in the general population and in patients with various clinical conditions (12).

PWV allows early identification of high-risk populations that may benefit from interventions to manage cardiovascular risk factors. Values greater than 10 m/s are already recognized as indicative of target organ damage, according to the main guidelines (15). A cross-sectional study conducted in Brazil between October 2018 and March 2019 demonstrated that a PWV above 8.2 m/s was more sensitive in the early detection of cardiovascular biomarkers associated with target organ damage (13).

It is known that a 1 m/s increase in PWV is associated with a 14% increase in the risk of cardiovascular events and a 15% increase in mortality. Although the clinical impact of PWV has been widely studied and recognized as a useful tool in the primary and secondary prevention of cardiovascular diseases, there are still significant challenges in its implementation in clinical practice (11).

### Fundus evaluation

The retina provides a direct view of the microcirculation, enabling detailed assessment of organic damage associated with cardiovascular diseases, such as arterial hypertension (16, 17). The retinal vascular system shares structural, functional and embryonic characteristics with the vessels of the heart, brain and kidneys, which makes the retina an important clinical window for the early detection of vascular alterations. Retinal alterations resulting from hypertension may reflect the vascular status of these internal organs, allowing for more accurate cardiovascular risk stratification, timely interventions and, consequently, an improved prognosis, highlighting its clinical importance (18).

The first evidence of ocular involvement by cardiovascular diseases dates back to 1836, when Richard Bright described its involvement associated with renal disease. After ratification of his discoveries with the advent of the ophthalmoscope in the mid-1850s, Liebreich described the first funduscopic alterations in malignant arterial hypertension, previously called Bright's disease. Hemorrhages, exudates and arteriolar abnormalities were already described at this time (19, 20). In 1898, Marcus Gunn described retinal changes in patients with cerebrovascular insufficiency and/or renal disease, such as narrowing and irregularities of the retinal arterioles (21). Spots seen in the fundus of patients with severe nephritis were observed in 1904 by Elschnig, characterizing the so-called Elsching spots, still known today (21). The first classifications of hypertensive retinopathy come from the work of Keith, Wagener and Barker (KWB), still in 1930. After this, several other classifications were proposed, such as the Waganer-Clay-Gipner and Scheie classifications (21). However, the KWB classification continues to be widely used to categorize retinal lesions associated with hypertension into four stages of severity. Other more recent classifications of malignant hypertension determine three categories of ocular involvement: hypertensive retinopathy, hypertensive choroidopathy and hypertensive optic neuropathy (21).

Hypertensive retinopathy progresses through three distinct phases. In the constrictive phase, there is a contraction of the retinal arterioles; in the sclerotic phase, there is thickening of the vascular wall; and, in the exudative phase, there is extravasation of fluids due to rupture of the retinal barrier (22). Given controlled systemic arterial pressure, vasoconstriction may disappear. Otherwise, the internal (endothelium) and external (retinal pigment epithelium) blood-retinal barriers are broken. With high and sustained blood pressure levels, there is an increase in vascular tone with a reduction in its lumen. The exudative phase progresses and includes retinal hemorrhages and cotton-wool exudates. Copper-wire vessels and silver-wire vessels represent the evolution of the process, characterizing the sclerotic phase. As this degenerative process progresses, hyalinization of the vascular wall occurs with loss of its muscle cells, resulting in various ocular complications related to the underlying disease, such as vascular occlusions and epiretinal membranes (21). Cohort studies indicate that changes in retinal vessels, especially arteriolar narrowing, may precede the development of hypertension and clinical cardiovascular diseases, reinforcing the relevance of fundus examination in hypertensive patients, as well as in individuals without diagnosed hypertension (23).

Evaluation of PWV (pulse wave velocity) in small arteries and microcirculation may be crucial for early detection of diseases, since endothelial dysfunction, often associated with vascular diseases, has a more significant impact on the microvasculature (24).

### Interrelationship between arterial stiffness, PWV and changes in the fundus

A growing number of studies have explored the relationship between large artery stiffness and retinal vasculature, highlighting the interaction between large and small arteries, using PWV as the primary tool to measure large artery stiffness (7, 10, 25, 26). Elevated PWV is closely associated with vascular aging and atherosclerosis, which also affects small ocular vessels. The ocular microcirculation, which includes the retinal and choroidal circulation, is sensitive to systemic hemodynamic changes, including those caused by arterial stiffness. In particular, the retinal vasculature is highly similar to that of other organs, such as the heart, brain, and kidneys, in terms of embryonic origin, anatomical characteristics, and pathophysiology (17).

In addition, the state of the systemic microcirculation is often assessed using the retinal arteries, which represent the only vascular system in the human body that can be directly observed (27). Thus, changes resulting from increased vessel stiffness may increase the risk of ocular diseases such as hypertensive and diabetic retinopathy, age-related macular degeneration (AMD), and glaucoma (28–31). Therefore, PWV may provide relevant information about the vasoregulatory capacity of the retina and possibly act as a biomarker for the onset and progression of retinal vascular diseases, which may serve as indicators to predict the risk of developing cardiovascular and cerebrovascular diseases (27, 31). In addition to ocular diseases, there are clear linear associations between morphometric measurements of retinal vessels and blood pressure and the arterial stiffness index. One such parameter studied is venular and arteriolar tortuosity. The association of increased tortuosity with higher blood pressure was independent of retinal diameters, suggesting that this assessment tool may offer additional value to cardiovascular risk prediction tools beyond current diameter tests (32, 33).

Furthermore, it is known that the relationship between retinal arteriolar narrowing and arterial stiffness can be quantified: for every 10 mmHg increase in systolic blood pressure, there is a 0.9 µm narrowing in arteriolar diameter. Therefore, narrower arterioles are associated with higher blood pressure, while wider venules are associated with inflammation and higher body mass index/obesity (32). Studies in the pediatric population have already demonstrated a possible positive association between obesity and changes in retinal vascular geometry, changes that appear to precede the development of apparent cardiovascular disease (34).

### Clinical implications

Hypertensive retinopathy plays a crucial role in risk stratification and prognostic value for the development of cardiovascular disease (25). The Nagahama study (28) demonstrated that narrowing of retinal arteries may be an early indicator of systemic atherosclerosis. Furthermore, retinal examination and measurement of retinal vascular diameters may benefit individuals with risk factors for cardiovascular and cerebrovascular disease by providing a noninvasive and accessible tool for early assessment (35). Clinical investigations have shown that individuals with hypertensive retinopathy have higher levels of PWV compared to those without the condition (2). Additional studies have shown a significant association between increased peripheral arterial stiffness and the severity of diabetic retinopathy, indicating that PWV may be a valuable indicator of microvascular disease progression in these patients (2, 36).

Arteriolar remodeling caused by hypertension can be assessed by analyzing retinal arterioles with state-of-the-art funduscopic cameras. However, this technology is not yet widely available. Recently, new retinal imaging techniques using smartphones have emerged as a promising alternative, allowing the assessment of hypertensive retinopathy in a larger number of patients (3). Currently, the assessment of retinal vascular changes is performed manually or semi-automatically, which can result in variability in measurements. Technical refinements are needed to minimize this variability and increase diagnostic accuracy (37).

In recent years, scanning laser Doppler and adaptive optics have been used to estimate the wall-to-lumen ratio of retinal arterioles. The wall-to-lumen ratio has been shown to be directly related to blood pressure load and other markers of hypertensive organ damage. In addition, it is correlated with structural changes in small arteries, measured by micromyography - the gold standard, albeit invasive, for assessing microvessels. While the prognostic value of changes in small subcutaneous arteries in hypertension has been documented, the predictive value of the retinal wall-lumen ratio and its variations throughout treatment remains to be established (3).

Investigating the structure and function of the microcirculation in the retina is essential to deepen the understanding of the pathogenesis of hypertension and its associated cardiovascular diseases. Compared to other imaging biomarkers used in the cardiovascular context, retinal imaging stands out for offering significant advantages, such as being a simple, fast, safe and cost-effective method. Furthermore, this technique provides essential information on target organ damage, which allows for accurate and differentiated risk assessment (37).

The retinal vasculature, therefore, emerges as an ideal site for the evaluation of microvascular changes in a noninvasive manner. With the use of technologies, it is possible to visualize and monitor these changes repeatedly and *in vivo*, which makes the retina a fundamental region in the assessment of vascular health and in the monitoring of therapeutic interventions (38).

### Limitations and challenges

Incorporating PWV into clinical practice allows for a more accurate assessment of arterial stiffness and, consequently, better management of conditions that affect cardiovascular health (39). At the same time, fundus examination can reveal vascular changes that are indicative of cardiovascular risk. Integrating these assessments allows for a more comprehensive approach to the patient, facilitating early identification of vascular changes and implementation of preventive strategies (33). Clinical guidelines recommend PWV assessment as an additional tool in the management of patients at high cardiovascular risk. The American Heart Association (AHA) and other organizations suggest that PWV be considered especially in patients with resistant hypertension, diabetes, or other cardiovascular risk factors (40). These recommendations aim to improve early detection and treatment of conditions that can lead to serious complications. Furthermore, recent studies recommend the integration of fundus examination and other ocular imaging methods in cardiovascular assessment, particularly in patients with hypertension and diabetes. Assessment of retinal vascular changes can provide valuable information about overall cardiovascular health and treatment efficacy (36).

Despite advances, studies on PWV and arterial stiffness face several limitations. A significant challenge is the variability in measurement methods and data interpretation. Studies show that different measurement techniques can result in significant variations in PWV results, which makes comparison between studies and uniform clinical application difficult (41–43). Furthermore, interpretation of PWV data can be complex and requires consideration of multiple factors, such as age, sex, and comorbidities (41, 44). Regarding fundus examination, although it is a powerful tool, its effectiveness can be limited by the examiner's skill and the quality of the equipment. Studies also indicate that early detection of vascular alterations can be challenging in the early stages of cardiovascular disease (45–47). One of the main challenges in clinical practice is the standardization of PWV and fundus assessment methods. The lack of uniform standards can lead to discrepancies in results and data interpretation. Standardization of measurement protocols and the adoption of uniform guidelines are essential to ensure the accuracy and reliability of assessments (48). Furthermore, the integration of these methods into daily clinical practices requires adequate training of professionals and continuous updating of the technologies and techniques used (45).

### Future perspectives

Arterial stiffness is an area of great scientific interest that promises to offer significant advances, especially with the hypothesis that vascular aging may not be inevitable. Measuring arterial stiffness through PWV remains a crucial technique for studying vascular aging. Based on this idea, there is the possibility that, in the future, specific therapeutic interventions could be developed to treat or even reverse this process, opening new perspectives for the prevention of cardiovascular diseases associated with human aging (49). cfPWV is the gold standard for assessing arterial stiffness in clinical practice. Several devices have been developed and validated to measure arterial stiffness noninvasively, using applanation tonometry (SphygmoCor, PulsePen), piezoelectric mechanotransducers (Complior), cuff-based oscillometry (Arteriograph, Vicorder and Mobil-O-Graph), photodiode sensors (pOpmètre) (50). Associated with these, new devices based on oscillometric detection of the brachial pressure waveform with a single cuff have been designed, free from the need to consider operator expertise and aiming to simplify measurement procedures and reduce delays (such as the BPLab and Mobil-O-Graph) (51).

In addition, increasing evidence suggests an association between systemic microvascular dysfunction and unfavorable cardiovascular outcomes. Fundus examination has emerged as a promising tool for noninvasive assessment of cardiovascular health, providing relevant information regarding microcirculation and arterial stiffness. The potential for using retinal vasculature as a marker of cerebrovasculature status offers clear advantages, due to the ease with which retinal vasculature can be directly visualized *in vivo*, and also photographed. With improvements in technology leading to a greater degree of automation and the development of real-time image analysis systems, it may be possible in the future to analyze a digital image of the retinal vasculature rapidly and obtain readily accessible information about an individual's potential risk of cerebrovascular disease (6).

## Conclusions

In this review, we demonstrated that systemic vascular diseases present an association between retinal arteriolar alterations and systemic vascular damage. An investigation based on fundus examination and PWV provides early identification of vascular alterations. Recognition of the ocular effects of blood pressure may allow better treatment and monitoring of its effects on target organs in these patients.

This review provides significant evidence that the use of the aforementioned methods can aid in clinical practice by stratifying disease risk as well as optimizing treatment, which can directly impact disease progression and, consequently, patients' quality of life. In addition, the implementation of standardized procedures is essential to ensure the clarity and credibility of data obtained from PWV and fundus examination assessments, and additional studies are needed to improve the application of these techniques.
